# The DD11 Material Components and Properties Impact and Relationship on Cutting Force in Progressive Stamping

**DOI:** 10.3390/ma19132806

**Published:** 2026-07-01

**Authors:** Juras Skardžius, Justinas Gargasas

**Affiliations:** Department of Mechanical and Materials Engineering, Faculty of Mechanics, Vilnius Gediminas Technical University, Saulėtekio al. 11, LT-10223 Vilnius, Lithuania; justinas.gargasas@vilniustech.lt

**Keywords:** material composition, material variables, part quality, statistical analysis

## Abstract

Progressive stamping is a high-efficiency sheet metal forming method in the automotive and mass production industries, where material characteristics significantly influence process stability, cutting force, tool life, and final part quality. Herein, we report the effects of the chemical composition and mechanical properties of DD11 low-carbon steel on punching force during progressive stamping. Ten DD11 material batches with varying chemical compositions and mechanical properties were subjected to experimental investigation. Material characterization involved spectroscopic chemical analysis, tensile testing in accordance with ISO 6892-1, and hardness measurement. Punching tests were performed with a Zwick BZ2-MMAG100.SH01 universal testing machine that incorporates a punch–die assembly to study force–displacement behavior under controlled conditions. The cutting curves of these materials were analyzed to determine the maximum cutting and fracture loads, which were then statistically correlated with the materials’ chemical and mechanical parameters. The results indicated that tensile strength and yield strength are the strongest statistically significant contributors to the maximum cutting load and the fracture point, and that the correlation coefficients for these measurements were +0.866 and +0.869, respectively. Carbon, chromium, and silicon showed the most positive effect on cutting resistance; whereas, titanium was negatively associated with each of the tested responses among chemical composition measures. But none of the chemical factors were statistically significant. The analysis also showed that material hardness yields the highest predictive performance for cutting force behavior (Pearson correlation coefficients up to 0.935 and a regression coefficient of R^2^ = 0.875). Results of this study show that DD11 cutting behavior at progressive stamping is controlled primarily by strength-dependent mechanical characteristics rather than chemical composition variations.

## 1. Introduction

Progressive stamping is considered one of the best manufacturing technologies in the automotive, electrical, and mass-production industries, as it enables high production rates, low cost, higher product yield, dimensional reproducibility, high accuracy, and fast throughput in sheet metal component fabrication [1,2,3]. This procedure incorporates different operations such as blanking, punching, piercing, bending, and forming into one die-oriented tool and, in turn, cuts the production price and improves the manufacturing productivity [2,4,5]. However, the efficiency and reliability of progressive stamping processes are strongly dependent on process stability, tool condition, and the stability of the material properties used, all of which greatly affect product quality in advanced stamping methods [6,7,8]. The key to this cutting and piercing issue lies in the interaction between the chemical composition and physical properties of the processed material [9,10]. Sheet metal can be extensively weakened by elastic–plastic deformation and crack initiation during a specific separation phase, followed by crack propagation [11]. Tensile strength, yield strength, hardness, ductility, strain hardening, and microstructural composition of the workpiece therefore directly influence the evolution of engineering cutting force [12,13,14]. The use of advanced, high-strength steels across industries has increased the need for a better understanding of the interactions between material characteristics and cutting performance [15,16]. Prior studies have shown a substantial increase in punching force and tool loading in stronger materials due to increased deformation and fracture resistance [17,18,19]. Mechanical properties of the material, e.g., hardness and tensile strength, significantly influence the burr, fracture morphology, sheared edge quality, and dimensional stability of the stamped components [13]. The chemical composition of a material is also fundamental to punching properties, since alloying elements influence strengthening mechanisms, microstructural stability, and fracture resistance [9]. The hardness, ductility, strain localization, and crack propagation properties of steel sheets have been investigated under the influence [11] of carbon, silicon, manganese, chromium, titanium, sulfur, and phosphorus. More generally, it has been shown that increasing the carbon and alloying contents generally increases power strength; whereas, simultaneously increasing cutting resistance increases blade wear [17]. Furthermore, sulfur inclusions and phosphorus segregation might lead to early crack initiation and decreased fracture stability during punching operations [10]. It is well established that part quality during progressive stamping depends heavily on material properties and process conditions. Material strength, thus, correlates with ductility and is directly associated with burr height, rollover zone, fracture surface morphology, and dimensional precision [1,2,7]. To assess the role of punching variables in shaping the sheared edge quality of ultra-high-strength steels, Mori and Abe [1] showed that improvements in punching parameters were substantial [14]. Highlighted here is the critical role of the material properties in determining blanking behavior and fracture evolution. Recent advances in manufacturing analytics have introduced state-of-the-art statistical methods for assessing manufacturing variability and process predictability [7,17,20]. Correlation models, Pareto-based evaluation, and machine learning methods are being applied to characterize key factors influencing cutting force, fracture point, and part quality in the sheet metal industry [8,18,19]. The aforementioned approach is considered particularly sensitive in progressive stamping, as multiple material parameters simultaneously affect the process response and tool loading conditions [21]. Although punching mechanics and sheet metal forming have been thoroughly investigated, few researchers have examined the quantitative relationships among material composition, mechanical properties, and cutting force performance in low-carbon structural steels used in industrial progressive stamping [10,11,14]. Additionally, the statistical significance of each alloying element and its relationship to the evolutionary punching force have not been well studied [17,21,22]. Consequently, this paper examines how the chemical composition of DD11 steel and its mechanical response influence the punching performance of progressive stamping processes. Cut tests were performed in the work using a universal testing system with a punch–die unit. Force–displacement characteristics were analyzed to determine the maximum cutting force and fracture point, while statistical correlation and Pareto-based methods were applied to determine the most influential material variables on cutting response and part quality.

## 2. Experimental Research Methodology

The research object was 10 industrial DD11 steel sheets from ten different production heat batches (heat numbers). The batches are provided by an industrial partner and cover typical variations in chemical composition and mechanical properties within the DD11 specification range. Using several heat batches was not to compare different steel grades, but to assess the influence of batch-to-batch variability inherent in a material grade on the cutting force response to progressive stamping.

Material chemical and mechanical properties analysis allows for the simultaneous study of the influence of several factors and their mutual interactions on cutting force. This is an important advantage for predicting the quality of upcoming parts and process stability. Conventional methods do not allow prediction and only observe the effect of the variable under study.

Experimental research was conducted to analyze how the mechanical and chemical properties of metallic materials influence cutting force during punching. Material sheet specimens were characterized by tensile testing, hardness measurements, and chemical spectroscopic analysis to determine their strength, ductility, and elemental composition. Actual material values (chemical composition and mechanical parameters) were acquired by manually testing each specimen. Material chemical composition was acquired by using the Oxford PMI Master PRO spectroscopic chemical analysis machine, and mechanical properties values were acquired by using the universal tensile testing machine—Zwick BZ2-MMAG100.SH01 (Zwick GmbH & Co. KG, Ulm, Germany), following the procedure according to the ISO 6892-1 standard [23].

**Figure 1:**
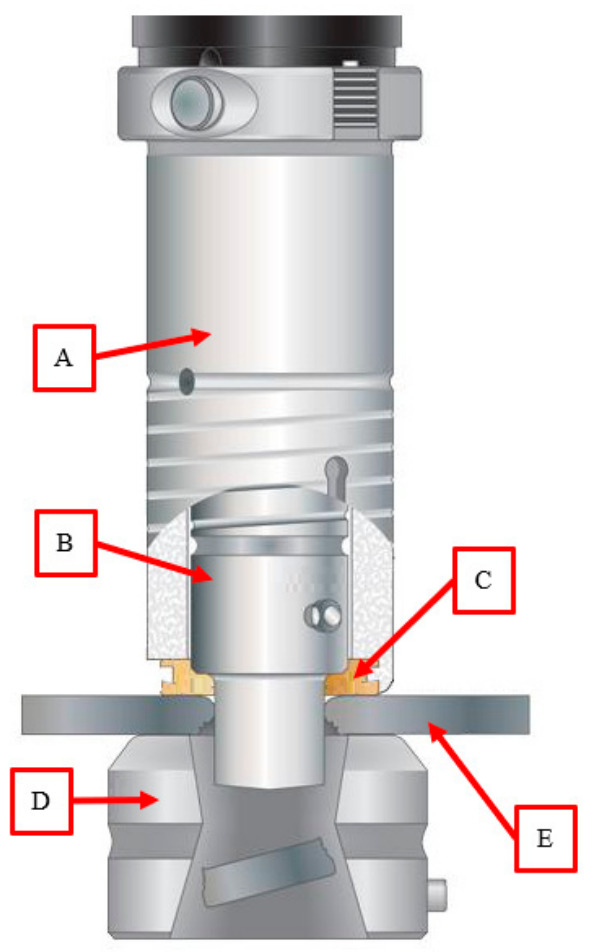
Set-up for test in tensile machine: (A) punch holder, (B) punch; (C) stripper; (D) die; (E) workpiece.

Punching experiments were performed using the same tensile testing machine—Zwick BZ2-MMAG100.SH01, adapted for cutting force measurement to simulate the loading conditions of progressive stamping. The tensile machine was equipped with a precision circular punch-and-die assembly (Figure 1), designed to ensure consistent alignment and clearance during testing. The punch was fabricated from high-speed tool steel (HSS) with a polished surface finish to minimize frictional effects, while the die block was hardened to resist deformation and wear.

The test specimens were flat metal sheets of uniform thickness (3.20 ± 0.02 mm) firmly clamped between the punch and die. Selected punch diameter was 10.1 mm. The die clearance was maintained at approximately 8% of the sheet thickness in order to ensure proper shearing without excessive burr formation. The chosen die clearance of 8% of the sheet thickness was based on industrial recommendations for low-carbon steel blanking processes, where typical clearance values are 5–10%. This range is known to strike a balance between cutting force stability, edge quality, and tool wear resistance. Clearance remained the same in each of the experiments in this study, so that differences in cutting force could only be explained by differences in material properties, not process parameters. Selected die diameter was 10.62 mm. The punching speed was held constant at 0.4 mm/s to enable quasi-static measurement of force development, and all tests were conducted under ambient laboratory conditions.

A load cell integrated with the tensile machine continuously recorded the applied force throughout the punching cycle, while the corresponding punch displacement was measured with high-precision linear encoders. The resulting force–displacement data were captured via the machine’s digital acquisition system, enabling accurate reconstruction of complete cutting curves for each tested specimen. Such testing under quasi-static conditions provides a reliable basis for comparing material performance and identifying relative trends; however, quantitative force levels and fracture behavior under real production conditions may differ, as high-speed industrial piercing may exhibit additional strain-rate, thermal, and frictional effects. Test parameters are presented in Table 1.

The typical force–displacement profile obtained during punching exhibits three distinct stages:

1. Elastic Deformation Stage: In the initial phase, the punch gradually contacts the sheet surface and elastically deforms it. The force increases almost linearly with displacement as the material resists initial indentation and bending. The slope of this linear segment reflects the material’s stiffness and elastic modulus.
2. Plastic Shearing Stage: As the punch penetrates further, localized plastic deformation initiates around the cutting edge. The force rises nonlinearly as shearing progresses through the sheet thickness. This stage dominates the overall cutting energy, with the magnitude of force depending strongly on the material’s yield strength, work hardening, and microstructural resistance to shear.
3. Fracture and Separation Stage: Once the material’s shear strength is exceeded, microcracks coalesce and propagate through the remaining section of the sheet. The force reaches its maximum just before complete separation, then drops rapidly as the blank detaches. The post-peak force decay rate provides insight into fracture mode: ductile materials show gradual force release; whereas, brittle materials exhibit a sudden drop.

**Figure 2:**
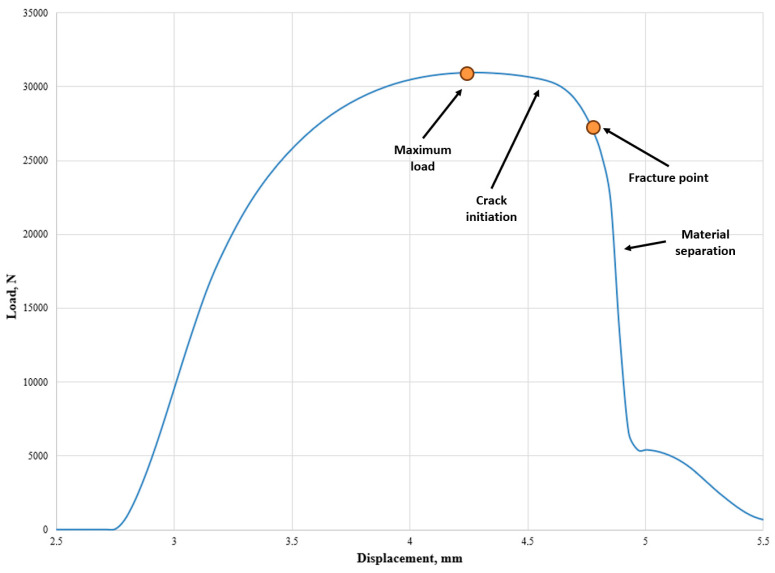
Piercing load curve stages.

For each material, the recorded cutting curve was analyzed to determine the maximum cutting force, cutting energy (area under the curve), and fracture displacement. For data extraction, maximum load was defined as the highest force value recorded during the punching cycle. The fracture load was defined as the force corresponding to the onset of unstable crack propagation, identified as the first point immediately after the maximum load at which the force–displacement curve showed a continuous decrease in force associated with material separation. Both values were extracted from the recorded force–displacement curves using the same procedure for all tested specimens. The load curve explanation is presented in Figure 2. These parameters were subsequently correlated with the corresponding mechanical and chemical properties to assess how intrinsic material behavior affects punching resistance. The reproducibility of the results was verified by repeating each test 10 times for each material type. For each specimen, the mean value, standard deviation (SD), and coefficient of variation (CV) were calculated from ten repeated measurements. These repeatability metrics were used to assess experimental consistency and measurement uncertainty prior to the statistical correlation analysis.

The results were evaluated using the Pareto principle as an analytical framework to assess the relative contribution of individual factors to the observed material behavior. The Pareto principle is an empirical heuristic stating that in complex systems, a limited number of variables are often responsible for the majority of measurable outcomes. Suggesting that approximately 20% of the processing parameters, variables, or microstructural features may account for nearly 80% of the variation in outcome results [24]. Data analysis by application of the Pareto principle enables the identification of the “vital few” contributors that exert the greatest impact on a given variable.

For each material batch, ten independent Vickers hardness measurements were performed. In addition to the arithmetic mean, the standard deviation (SD) and coefficient of variation (CV) were calculated to evaluate within-specimen variability and measurement repeatability.

## 3. Results

**Figure 3:**
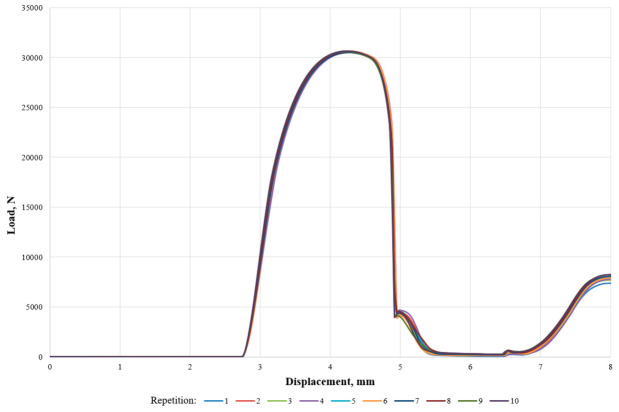
Specimen JF5995, 10 repetition graphs.

**Figure 4:**
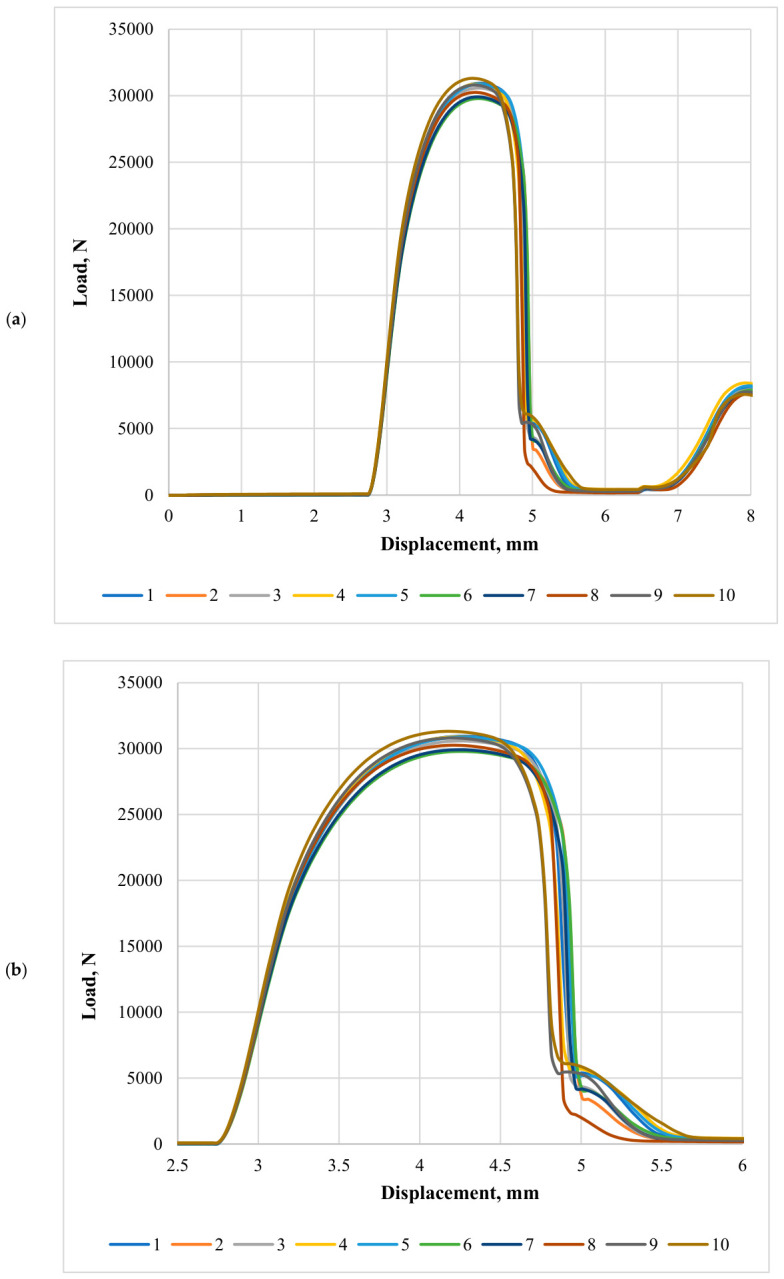
All specimens’ average values graphs: (**a**) full graph view; (**b**) zoomed piercing operation view.

**Figure 5:**
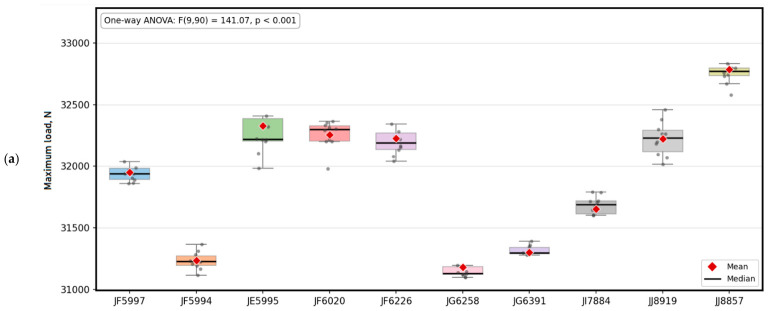

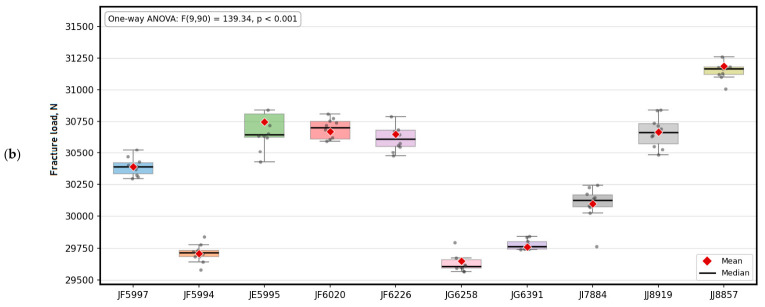
Recorded values distribution view: (**a**) maximum load distribution; (**b**) fracture load distribution.

For material parameter evaluation, 10 pieces of material with different compositions were selected. Material properties are presented in Table 2. To prevent any impact from prior test runs and minimize the risk of further errors, the setup was thoroughly disassembled and cleaned after each test. Each specimen was tested 10 times. The results were statistically processed and expressed as mean ± standard deviation (SD). Additionally, the coefficient of variation (CV) was calculated to quantify the dispersion of the data. A sample of the 10 repetitions of data is presented in Figure 3, and all specimens’ average value graphs are presented in Figure 4. After the tests were performed, maximum load and fracture load values were extracted for further calculations. Test key data are presented in Table 3, and the distribution of measured values is shown in Figure 5.

The enlarged view highlights the characteristic region used to extract the maximum load and fracture load values. Labels and annotations were added to improve readability and facilitate interpretation of the force–displacement response.

Repeatability analysis of both the maximum load and fracture load measurements revealed limited variability within batches. The calculated coefficient of variation was low for all tested specimens, suggesting stable experimental conditions and good measurement consistency. Also, variability between batches of different materials increased relative to the variability captured by repeated measurements within the same batch, thereby validating the subsequent correlation analyses.

Until the displacement reaches around 2.7 mm, the measured load remains close to zero, indicating that the tool is approaching the point of initial contact with the specimen without any material deformation. Afterward, a sharp rise in load begins, marking the onset of plastic deformation and shear zone development beneath the punch. The load increases steeply with displacement, reaching a maximum load from 29,788.7 N of specimen No. 6 to 31,311.1 N of specimen 10, and the maximum load value shifts from 4.16 mm to 4.32 mm. As the DD11 material is ductile low-carbon steel, the distinct elastic–plastic transition point is not clearly visible during piercing. Immediately after the peak, a sudden and pronounced drop in load is observed, indicating complete fracture and breakthrough of the material. Following the fracture, the load decreases rapidly to near-zero values as the punch passes through the sheet, with the resistance largely relieved. At larger displacements, from around 6.5 mm to 8 mm, a secondary increase in load is observed, rising gradually to approximately 8000 N by 8 mm displacement. This post-fracture load is attributed to contact between the punch and the scrap from the test before.

As elastic–plastic deformation point was not clearly visible and only could be approximately calculated, this limitation affected the identification of the elastic–plastic transition only and did not affect the extraction of maximum load and fracture load values, which were identified directly from the recorded force–displacement curves, it was decided to determine relationship between material properties and maximum load value and material breakage points by separating results into the following group:

1. The influence of material chemical composition on maximum load;
2. Material chemical composition at the fracture point;
3. Material mechanical properties on the maximum load;
4. The material mechanical properties influence the fracture point;
5. The material chemical and mechanical properties influence the maximum load;
6. Material chemical and mechanical properties influence the material fracture point.

To correctly define the research findings, a statistical significance coefficient was used. This allows us to ensure that the results can be trusted and that the vital few factors are separated from the insignificant many. The importance of this threshold is reflected in how the analysis is interpreted: significant factors have a strong and reliable impact on material performance, while non-significant factors are considered to have weak or negligible influence. The calculated statistical significance coefficient was shown as a red dotted reference line on each result chart, and bars crossing this line indicate factors with a statistically significant impact.

Statistical significance coefficient $r_{\mathrm{critical}}$, was calculated by the following formula:

(1) $$r_{\mathrm{critical}}=\frac{t_{\mathrm{critical}}}{\sqrt{t_{\mathrm{critical}}^{2}+df}}$$

where r is the correlation coefficient; $t_{\mathrm{critical}}$ is the t-value derived from statistical tables based on the sample size (*n* = 10), with $t_{\mathrm{critical}}=2.306$ for a two-tailed test at a 5% significance level; df is the degrees of freedom, which for n=10 is 8.

Using this formula, the critical value for the correlation coefficient $|r_{\mathrm{critical}}|$ at n=10 is determined to be 0.632. This means that for a correlation to be deemed statistically significant (i.e., indicating a meaningful relationship), its absolute value must exceed 0.632. Correlations below this threshold are not considered statistically significant, as they could be attributed to random chance.

**Figure 6:**
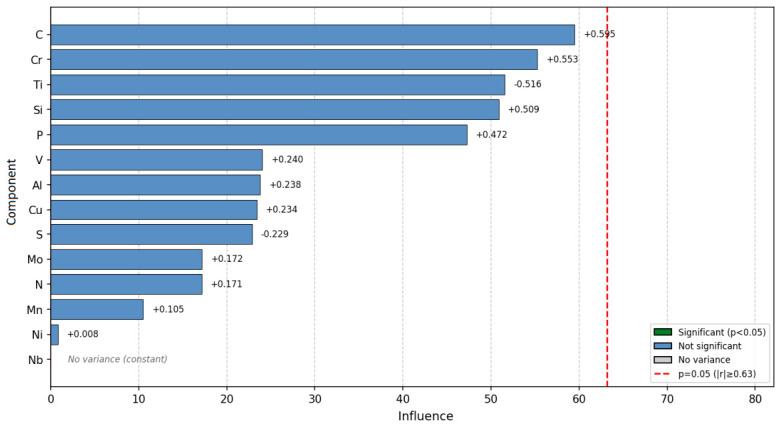
The influence of the material chemical composition on the maximum load.

**Figure 7:**
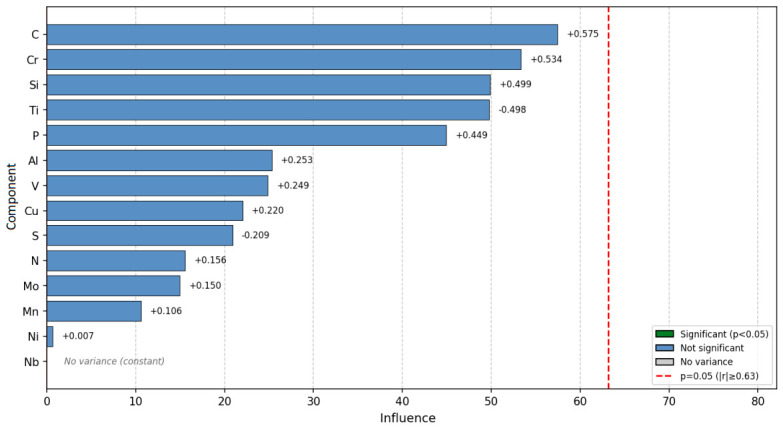
The influence of material chemical composition on the fracture point.

**Figure 8:**
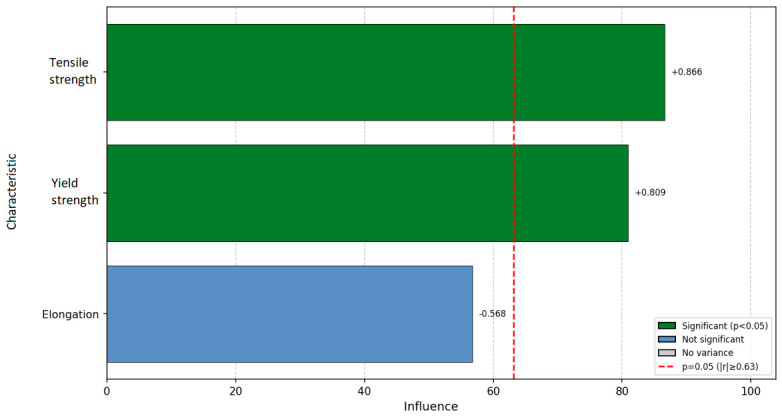
The influence of material mechanical properties on the maximum load.

**Figure 9:**
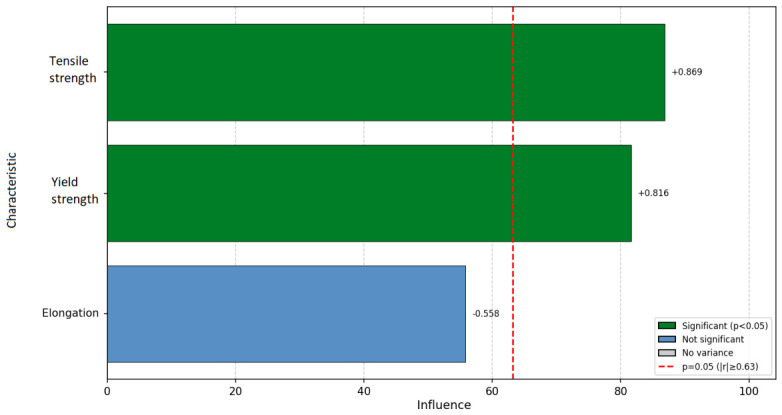
The influence of material mechanical properties on the fracture point.

**Figure 10:**
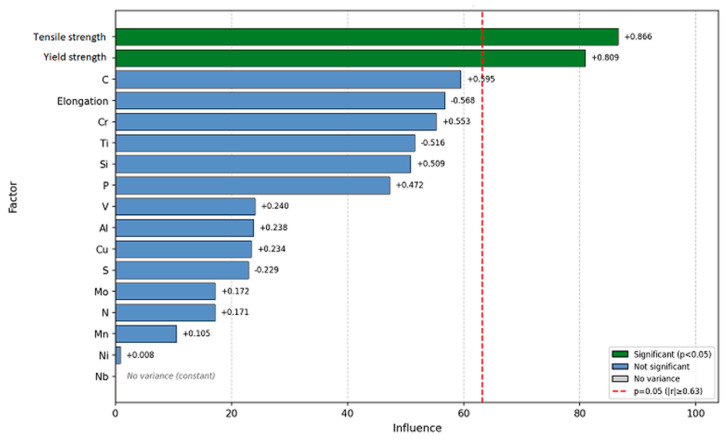
The influence of material chemical and mechanical properties on maximum load.

**Figure 11:**
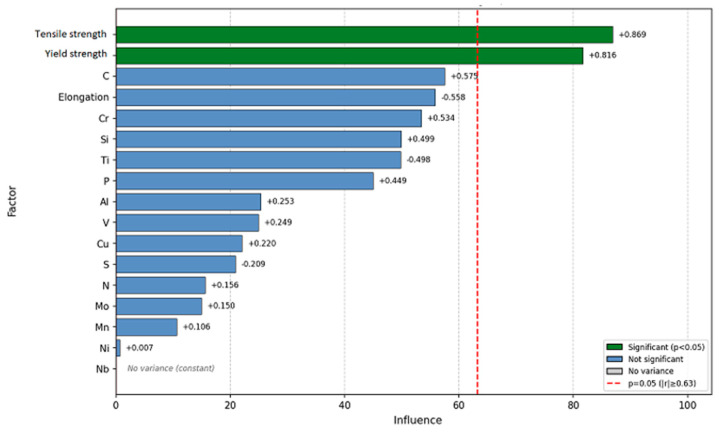
The influence of material chemical and mechanical properties on the material fracture point.

After processing the data using the statistical program Minitab 2.0, the results are presented in Figure 6, Figure 7, Figure 8, Figure 9, Figure 10 and Figure 11.

The correlation-based Pareto analysis reveals that, among all chemical constituents, carbon exhibits the strongest positive correlations with both the maximum cutting load (+0.595) and the fracture point (+0.575), highlighting its central role in increasing strength and resistance to deformation during cutting. Chrome and silicon also show consistently strong positive influences, reflecting their contributions to solid-solution and carbide-related strengthening mechanisms that improve load-bearing capacity up to the point of fracture. In contrast, titanium displays a clear negative correlation with both responses, with coefficients of −0.516 for the maximum load and −0.498 for the fracture point. These results indicate that increasing titanium content may be associated with a reduced ability of the material to sustain high cutting forces. However, the underlying mechanisms cannot be confirmed solely from the present data. Phosphorus contributes positively to load capacity and rupture resistance but remains a critical element due to its well-known tendency to promote brittleness. Sulfur shows a modest but consistently negative effect, likely associated with inclusions-driven crack initiation during cutting. Other elements such as vanadium, aluminum, copper, nickel, manganese, molybdenum, and nitrogen exert only minor influence within the investigated composition range, while niobium shows no measurable effect due to its constant concentration across the specimens. Overall, the results indicate that cutting behavior is primarily strength-controlled, with similar chemical drivers influencing both the maximum load and the fracture point, emphasizing the importance of carefully balancing strengthening elements against embrittling constituents to optimize cutting performance. Yet none of the chemical components’ influence was recognized as significant.

It should be noted that the present study was based on only ten independent material batches (*n* = 10), resulting in a relatively high critical correlation coefficient (|*r*| = 0.632 at α = 0.05). Consequently, only very strong relationships could be identified as statistically significant. Therefore, the absence of statistically significant correlations for chemical composition variables should not be interpreted as conclusive evidence that these factors have no influence on cutting behavior. Moderate chemical effects may remain undetected because of limited statistical power, leading to a potential Type II error. Future investigations involving larger numbers of material batches are required to verify the observed trends and improve sensitivity to smaller effect sizes.

However, statistical significance must not be confused with practical significance. Some chemical composition variables showed effect sizes that would likely be practically relevant according to Cohen’s common effect size guidelines (*r* = 0.10: small, *r* = 0.30: medium, *r* = 0.50: large). Specifically, carbon was correlated with maximum load (*r* = 0.595) and fracture load (*r* = 0.575), indicating large effect sizes despite not exceeding the statistical significance threshold. As such, these findings should be interpreted as potentially relevant trends and confirmed by examining studies with larger sample sizes, rather than by evidence of little impact.

Evaluating the influence of material mechanical properties on the maximum load value, tensile strength emerges as the most influential parameter, exhibiting a very strong and statistically significant positive correlation of +0.866. This suggests that increases in tensile strength directly and substantially enhance the load-bearing capacity during cutting. Yield strength follows closely, also showing a strong and significant positive correlation of +0.809. This confirms that resistance to plastic deformation is a key factor controlling the peak cutting load, as materials with higher yield strength require greater force to initiate and propagate plastic flow under the cutting tool. Elongation, in contrast, shows a moderate negative correlation of −0.568 that does not reach statistical significance, indicating that ductility plays a secondary, less consistent role in determining the maximum load.

A very similar trend is observed in the results for the fracture point, which characterizes the material’s resistance before breaking. Tensile strength again shows the strongest influence, with a strong and statistically significant positive correlation of +0.869, indicating that materials with higher ultimate strength fracture at higher loads during cutting. Yield strength remains a critical contributor as well, displaying a significant positive correlation of +0.816, reinforcing the idea that both the elastic–plastic transition and ultimate strength govern fracture resistance. As with maximum load, elongation exhibits a moderate negative correlation of −0.558 that does not exceed the significance threshold, implying that increased ductility may slightly reduce fracture load but does not decisively control failure under the investigated conditions.

When these two analyses are considered together, they demonstrate a coherent mechanical interpretation: the cutting response of DD11 steel is predominantly controlled throughout the entire loading process, from peak load to final fracture. Tensile and yield strengths consistently exceed the statistical significance threshold and account for the majority of the influence on both maximum and fracture loads, while elongation plays a subordinate and statistically uncertain role. This suggests that, for cutting performance optimization and fracture resistance in DD11, controlling strength-related properties is far more critical than modifying ductility within the examined material range.

The key differences between the separate and combined chemical and mechanical properties lie in the specific material properties that correlate with the maximum load and fracture point. While tensile and yield strength have a significant impact in both cases, the presence of certain elements such as carbon, chrome, and titanium in the analyses demonstrates that chemical composition plays a slightly more prominent role than, or the same role as, a material elongation parameter in both the maximum load and fracture point cases.

**Figure 12:**
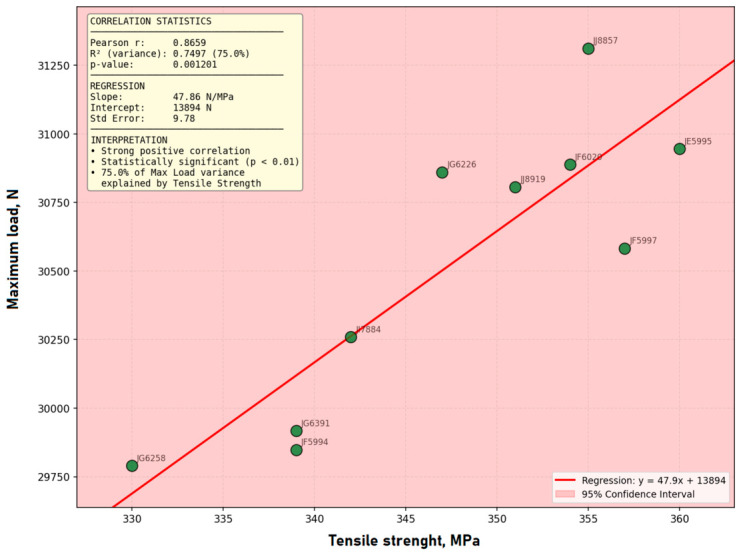
Correlation between tensile strength and maximum load.

**Figure 13:**
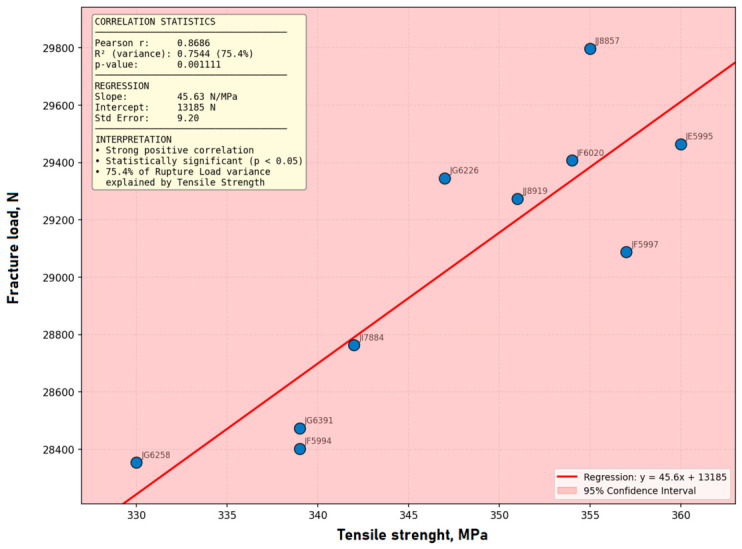
Correlation between tensile strength and material fracture point.

As tensile strength has the greatest influence on the maximum load and the material’s fracture point, a correlation between these characteristics was established. The data were evaluated using the statistical software MINITAB 2.0. For the correlation, a 95% confidence interval was selected. The results are presented in Figure 12 and Figure 13.

The Pearson correlation coefficient indicates how strongly two variables are linearly related and ranges from –1 to +1. Pearson correlation coefficient for tensile strength and maximum load was *r* = 0.8659, and for tensile strength and material fracture point was *r* = 0.8686. Results indicate a strong linear relationship, with the trust factor exceeding 86.5% for both correlations. Additionally, the statistical significance is very low in both cases, indicating that the pattern observed in the data is very unlikely to be due to random chance. The calculated statistical significance value between tensile strength and maximum load is *p* = 0.001201, and the statistical significance value between tensile strength and material fracture point is *p* = 0.001111.

The calculated regression coefficient between tensile strength and maximum load is R^2^ = 0.7497, and the correlation coefficient between tensile strength and material fracture point is R^2^ = 0.7544. Results show that material fracture points offer greater dependence and predictability than maximum load values, but because fracture point extraction is more complex, they are more difficult to use in real-life applications, as more complex algorithms must be used compared to maximum load extraction algorithms. Nevertheless, regression results for both cases suggest that predictability is viable, but predicted results may differ from real process data.

**Figure 14:**
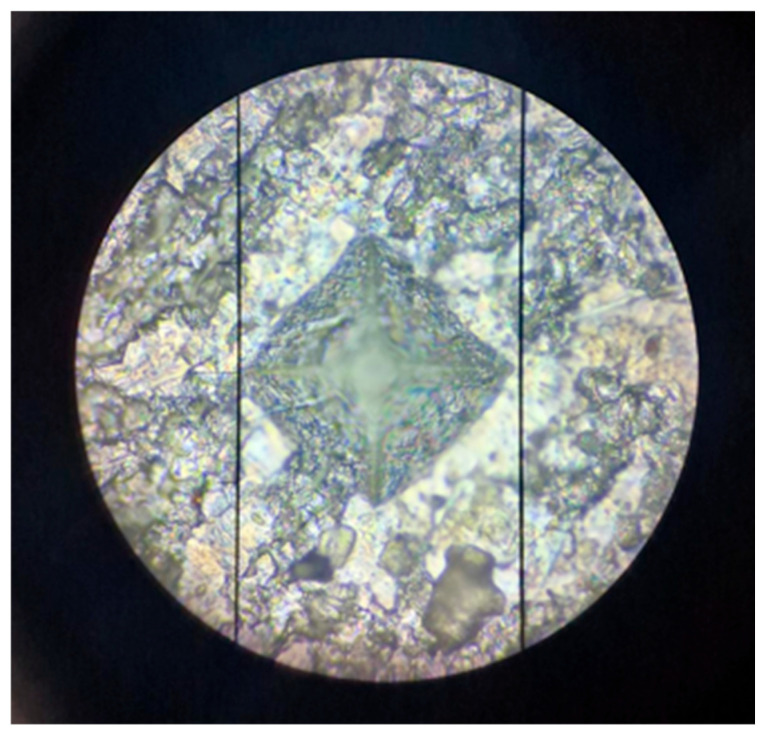
Hardness measuring diamond indentation.

As tensile strength shows the maximum stress that a material can withstand before breaking, this characteristic is closely related to material hardness, which indicates resistance to localized plastic deformation [25]. As this relationship is indirect rather than strictly linear, an additional correlation between previous test maximum values, material breakage points, and sample hardness was decided to be performed. Material hardness was measured with the Mitutoyo MH-200 machine (Mitutoyo, Kawasaki, Japan). Hardness was measured by pressing a diamond indenter (a pyramid with an angle of 136° between opposite faces) into the material and measuring the diagonals of the indentation, as shown in Figure 14. For measurements, 50× magnification was used. Test parameters are presented in Table 4.

**Figure 15:**
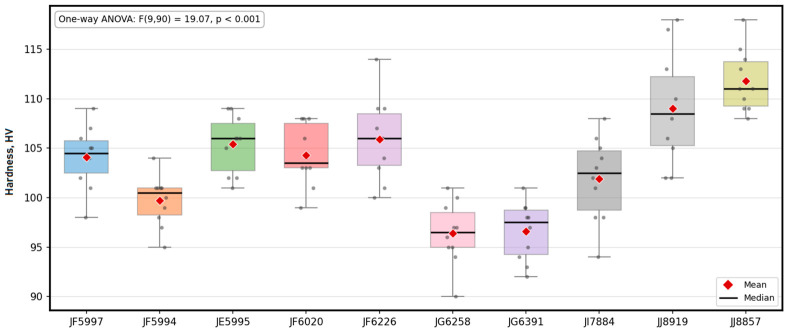
Recorded material hardness values distribution view.

For each specimen tested for hardness, 10 repetitions were performed, and the final result was taken as the test’s average value. Test results are presented in Table 5, and the distribution of recorded values is shown in Figure 15.

The calculated standard deviations and coefficients of variation for hardness indicated relatively low within-specimen variability. Although individual hardness measurements exhibited local fluctuations, these variations are expected in industrial sheet steels due to microstructural heterogeneity, grain-orientation effects, and the localized nature of indentation testing. The observed variability did not compromise the reliability of the calculated average hardness values used in the correlation analysis.

Ten hardness measurements per specimen were considered sufficient because the resulting average values exhibited stable trends and low coefficients of variation. Increasing the number of indentations would improve statistical precision but is unlikely to substantially alter the observed relationships between hardness and punching force.

**Figure 16:**
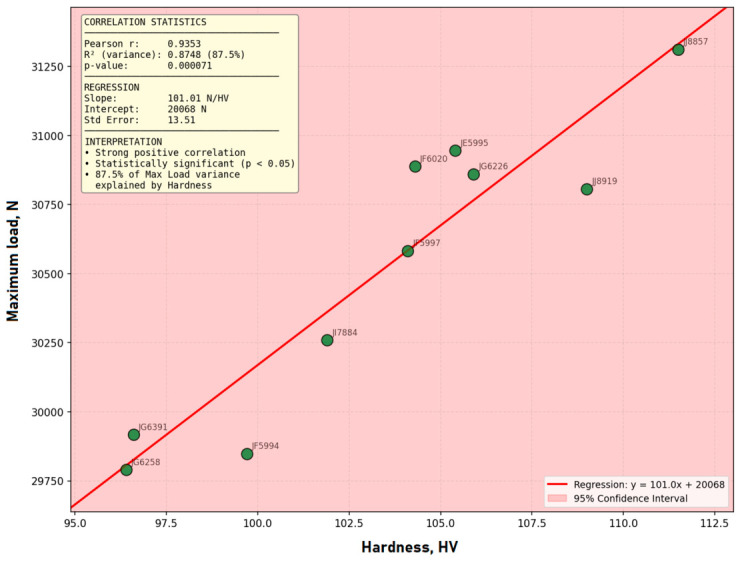
Correlation between material hardness and maximum load.

**Figure 17:**
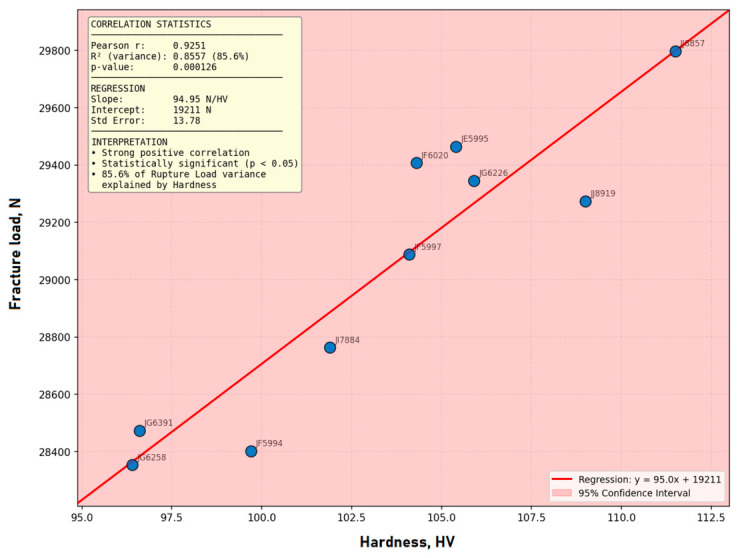
Correlation between material hardness and material fracture point.

As with tensile strength, a correlation between the maximum value and the fracture point was established. Correlation was performed using the same parameters and statistical program as before. The results are presented in Figure 16 and Figure 17.

Based on the experimental data, a linear regression model was developed to describe the relationship between hardness (HV) and maximum cutting force (Fmax):

*Fmax* = *a*·*HV* + *b* (2)

where *F_max_* is the maximum cutting force (N), and *HV* is Vickers hardness.

The obtained regression model shows a strong correlation (R^2^ = 0.8748), indicating high predictive capability within the tested range.

The Pearson coefficient test results show even greater values than the material tensile strength correlation. Pearson correlation coefficient for hardness and maximum load was *r* = 0.9353, and for hardness and material fracture point was *r* = 0.9251. The same tendency could be observed with a statistically significant value. Between hardness and maximum load, *p* = 0.000071, and the statistical significance value between hardness and material fracture point is *p* = 0.000126.

Finally, the hardness regression coefficient exceeds that of both tensile strength regression models, indicating stronger dependence and predictability. The calculated regression coefficient between hardness and maximum load is R^2^ = 0.8748, and the correlation coefficient between hardness and material fracture point is R^2^ = 0.8557. Compared with tensile strength regression, coefficients increased by 0.1251 for the maximum load value and 0.1013 for the fracture point, allowing for more precise data to be obtained more quickly.

## 4. Discussion

Findings indicate that most of the punching force behavior in DD11 steel is attributable to mechanical characteristics, specifically tensile strength, yield strength, and hardness. These parameters showed strong, statistically significant correlations with the maximum cutting load and the fracture load. By comparison, none of the chemical composition variables examined exceeded the threshold for statistical significance. But the interpretation of non-significant chemical effects is less clear-cut. This analysis was performed using only 10 independent batches of materials, yielding 8 degrees of freedom and a relatively high critical correlation coefficient (|*r*| = 0.632). With these parameters, only strong correlations can be properly reported, and moderate effects may be less detectable. Hence, the limited strength of statistical correlations among the chemical constituents should be considered partial evidence for the absence of a significant influence of chemical composition on punching behavior. Some chemical elements, in particular carbon, chromium, and silicon, showed modest increases in their positive correlation with cutting resistance and the metallurgical contribution and thus may still be of practical importance. These observations are consistent with the well-established functional role of alloying elements in both strengthening mechanisms and fracture resistance. It is therefore necessary to further research these trends using larger datasets and greater compositional variation to ascertain whether these observations are indeed material effects. Statistical aspects would likely greatly improve the detection of medium effect size and decrease the Type II error probability with larger sample sizes. Further studies with more than 30 independent material batches could enable a more robust investigation of the effects of chemical composition and the generalization of predictive models to progressive stamping applications.

For this study, the distinction between statistical significance and effect size is particularly important. None of the chemical composition variables under investigation reached statistical significance, but many produced moderate-to-large correlation coefficients. Carbon, for example, demonstrated a correlation coefficient approaching the significance threshold (*r* = 0.595; *r* = 0.595 is consistent with a large effect size, according to Cohen’s classification). Cohen’s interpretation of the influence on the tested material properties is presented in Table 6.

In the absence of a statistically significant relationship, this finding suggests the lack of significance may stem from small sample sizes rather than the absence of a practically meaningful relationship. As such, chemical composition effects should be considered preliminary trends needing to be validated on larger experimental datasets.

It should be noted that the chosen clearance is important in determining the absolute magnitude of the cutting force, but because it is constant across all tests, it does not influence the correlations between cutting response and material properties.

The inclusion of SD and CV ensures compliance with standard experimental data reporting practices in materials testing and improves comparability with similar studies.

The linear relationship between mechanical properties (hardness, tensile strength, and yield strength) and cutting force was attributed to resistance to plastic deformation and fracture mechanisms. Three stages (elastic deformation, plastic shearing, fracture) are achieved in the cutting process during progressive stamping. The plastic shear stage is where the maximum cutting force occurs, and dislocation motion and local shear band formation dictate the material response. Therefore, a high-yield-strength material requires a high applied stress to initiate plastic deformation; whereas, higher tensile-strength materials resist the initiation and growth of cracks at the ultimate fracture stage. Hardness is defined as resistance to localized plastic deformation as measured by the strain indentation and shear under punch loading. Thus, an increase in hardness directly results in higher cutting resistance and peak force values. On the other hand, the chemical composition does not directly control immediate mechanical resistance during cutting but indirectly influences it through microstructural strengthening mechanisms.

## 5. Conclusions

This research indicated a relationship between the material, chemical, and mechanical properties and the influence on piercing force and fracture point. After conducting research, the following conclusions were formulated:

1. Of all the determined mechanical properties, tensile strength was found to have the most consistent and statistically significant correlation with maximal cutting load and fracture load, revealing that resistance to plastic deformation acts as the primary governing force for the development of cutting force. The inclusion of the standard deviation and the coefficient of variation confirms that the observed trends are not influenced by excessive experimental scatter, thereby supporting the robustness of the correlation analysis.
2. Hardness had the best relationship with cutting force reaction and fracture characteristics, which indicates that hardness has the potential to be a useful and optimal determination of the material resistance of DD11 steel under the progressive stamping analysis.
3. Taken together, the examined chemical elements were not statistically significant within this dataset; however, moderate-to-large effect sizes for carbon, chromium, and silicon imply practical relevance in the field of cutting resistance. Such relationships should accordingly be interpreted as indicative trends that require validation in larger sample populations.
4. Titanium showed a consistent negative relation to both maximum load and fracture load, which is an important observation to consider, as the effect on microstructural features that had an effect on deformation and fracture behavior requires further investigation.
5. The synthesis of chemical and mechanical parameters indicates that mechanical characteristics mainly describe the variability of the cutting force performance, but it is the composition of the material that may indirectly affect the cutting force.
6. Overall, the findings suggest that it is feasible, under the experimental conditions, to approximate the cutting force and fracture behavior of DD11 steel with the aid of mechanical property-based indicators like hardness and tensile strength. The results can be used to inform material selection and cutting force estimation for progressive stamping procedures, while larger material datasets and industrial conditions have also been investigated to improve generalization.
7. A limitation of the present study is the relatively small number of investigated material batches (n = 10). While this sample size was sufficient to identify strong correlations between mechanical properties and cutting-force responses, it limited the ability to detect moderate effects of chemical composition. Therefore, non-significant chemical correlations should be interpreted cautiously, and future studies should include larger datasets (n ≥ 30) to improve statistical power and validate the observed trends.

## Figures and Tables

**Table 1:** Test parameters.

Characteristic	Value
Pre-load	65 N
Test speed	0.4 mm/s
Tool separation	20 mm
Distance till billet	8.7 mm

**Table 2:** Tested specimen properties.

Specimen No.	Material Heat No.	Al, wt%	C, wt%	Cr, wt%	Cu, wt%	Mn, wt%	Mo, wt%	N, wt%	Nb, wt%	Ni, wt%	P, wt%	S, wt%	Si, wt%	Ti, wt%	V, wt%	Elongation, %	Tensile Strength, MPa	Yield Strength, MPa
1	JF5997	0.029	0.049	0.028	0.013	0.263	0.002	0.003	0.003	0.029	0.009	0.002	0.041	0.01	0.002	45.4	357	266
2	JF5994	0.04	0.039	0.018	0.008	0.22	0.001	0.002	0.003	0.017	0.007	0.008	0.015	0.011	0.001	47.9	339	251
3	JE5995	0.034	0.043	0.02	0.008	0.222	0.001	0.004	0.003	0.018	0.009	0.006	0.016	0.011	0.002	47.3	360	270
4	JF6020	0.041	0.044	0.024	0.013	0.232	0.001	0.001	0.003	0.02	0.009	0.01	0.02	0.012	0.001	45	354	266
5	JF6226	0.036	0.049	0.021	0.008	0.246	0.001	0.001	0.003	0.018	0.009	0.008	0.016	0.012	0.001	47.3	347	255
6	JG6258	0.032	0.043	0.021	0.01	0.239	0.001	0.001	0.003	0.019	0.009	0.007	0.01	0.013	0.001	46.9	330	235
7	JG6391	0.033	0.044	0.02	0.006	0.211	0.001	0.001	0.003	0.012	0.008	0.006	0.016	0.011	0.001	46.7	339	244
8	JI7884	0.024	0.043	0.023	0.012	0.203	0.001	0.003	0.003	0.016	0.011	0.004	0.008	0.009	0.001	47.2	342	251
9	JJ8919	0.036	0.05	0.03	0.012	0.221	0.002	0.004	0.003	0.016	0.011	0.003	0.034	0.003	0.001	43.7	351	254
10	JJ8857	0.036	0.047	0.027	0.009	0.215	0.001	0.001	0.003	0.012	0.010	0.003	0.030	0.003	0.001	44.6	355	264

**Table 3:** Tests key data.

Specimen No.	Material Heat No.	Maximum Load, N	Maximum Load Displacement, mm	Standard Deviation	Coefficient of Variation (%)	Fracture Load, N	Fracture Load Displacement, mm	Standard Deviation	Coefficient of Variation (%)
1	JF5997	30,582.9983	4.260521977	81.8	0.26	29,088.03552	4.691914829	71.2	0.23
2	JF5994	29,848.2127	4.606121036	72.2	0.23	28,401.85124	5.064211342	70.4	0.24
3	JE5995	30,945.660	4.265059615	275.9	0.85	29,464.08076	4.672627957	271.1	0.88
4	JF6020	30,887.7187	4.242153844	114.9	0.36	29,407.06513	4.652014312	122.0	0.40
5	JG6226	30,860.6257	4.325972991	169.2	0.53	29,344.94343	4.763523391	165.1	0.54
6	JG6258	29,790.2072	4.320285752	111.4	0.36	28,353.99851	4.778064055	104.8	0.35
7	JG6391	29,916.8346	4.350087581	69.6	0.22	28,472.99822	4.789196661	71.8	0.24
8	JI7884	30,259.1733	4.395484969	145.5	0.46	28,763.32002	4.864661358	136.3	0.45
9	JJ8919	30,806.9367	4.514744032	139.7	0.43	29,273.6231	4.897488883	122.3	0.40
10	JJ8857	31,311.9711	4.825249953	155.7	0.47	29,797.44485	5.224247055	145.5	0.47

**Table 4:** Hardness measurement test parameters.

Characteristic	Value
Load time	4 s
Test time	10 s
Unload time	4 s
Test speed	60 μm/s
Force mode	1

**Table 5:** Material hardness measurement results.

Specimen No.	Material Heat No.	Measured Hardness, HV										Average Value	Standard Deviation	Coefficient of Variation (%)
		1	2	3	4	5	6	7	8	9	10			
1	JF5997	101	98	105	104	102	109	105	107	106	104	104.1	3.1	3.02
2	JF5994	101	100	101	99	98	101	101	104	95	97	99.7	2.5	2.55
3	JE5995	102	109	106	108	106	102	101	105	109	106	105.4	2.9	2.76
4	JF6020	103	101	108	106	103	108	99	104	103	108	104.3	3.1	3.00
5	JF6226	103	109	106	107	101	104	109	114	106	100	105.9	4.2	3.94
6	JG6258	95	99	100	94	90	97	95	97	101	96	96.4	3.2	3.32
7	JG6391	98	99	92	97	95	93	98	101	94	99	96.6	3.0	3.06
8	JI7884	101	94	108	98	102	103	106	104	105	98	101.9	4.3	4.18
9	JJ8919	109	102	118	117	102	113	110	108	106	105	109	5.6	5.17
10	JJ8857	113	109	114	109	118	108	110	111	115	111	111.8	3.2	2.82

**Table 6:** Cohen influence interpretation.

Variable	*r*	Cohen Interpretation
Carbon	0.595	Large
Elongation	0.558	Large
Chromium	0.55	Large
Silicon	0.50	Large
Titanium	−0.516	Large (negative)

## Data Availability

The original contributions presented in this study are included in the article. Further inquiries can be directed to the corresponding author.
